# Mental health impairment in underweight women: do body dissatisfaction and eating-disordered behavior play a role?

**DOI:** 10.1186/1471-2458-11-547

**Published:** 2011-07-10

**Authors:** Jonathan Mond, Bryan Rodgers, Phillipa Hay, Cathy Owen

**Affiliations:** 1 School of Sociology, Australian National University, Canberra, Australia; 2 Australian Demographic & Social Research Institute, Australian National University, Canberra, Australia; 3 School of Medicine, University of Western Sydney, Campbelltown, Australia; 4 Rural Clinical School, Medical School, Australian National University, Canberra, Australia

**Keywords:** Body dissatisfaction, eating-disordered behaviour, mental health, underweight, women

## Abstract

**Background:**

We sought to evaluate the hypothesis that mental health impairment in underweight women, where this occurs, is due to an association between low body weight and elevated levels of body dissatisfaction and/or eating-disordered behaviour.

**Methods:**

Subgroups of underweight and normal-weight women recruited from a large, general population sample were compared on measures of body dissatisfaction, eating-disordered behaviour and mental health.

**Results:**

Underweight women had significantly greater impairment in mental health than normal-weight women, even after controlling for between-group differences in demographic characteristics and physical health. However, there was no evidence that higher levels of body dissatisfaction or eating-disordered behaviour accounted for this difference. Rather, underweight women had significantly lower levels of body dissatisfaction and eating-disordered behaviour than normal-weight women.

**Conclusions:**

The findings suggest that mental health impairment in underweight women, where this occurs, is unlikely to be due to higher levels of body dissatisfaction or eating-disordered behaviour. Rather, lower levels of body dissatisfaction and eating-disordered behaviour among underweight women may counterbalance, to some extent, impairment due to other factors.

## Background

In the past decade, there has been considerable interest in the question of whether, and how, obesity might affect individuals' mental health, both the occurrence of specific psychopathology [1-7] and the perceived effects of psychopathology on psycho-social functioning [8-14]. Conclusions in this regard have been found to vary according to the study design, the characteristics of the population sampled and the measures of mental health employed [5-7,15-17]. However, one finding that has been consistently reported is that obesity is more likely to be associated with mental health impairment in women than in men [1-3,5-7,10,13-18]. Further, evidence suggests that this disparity reflects, at least in part, the fact that the prevalence of body dissatisfaction and eating-disordered behaviour is higher - and the adverse effect of these variables on mental health greater - in overweight women than in overweight men [19,20].

Interestingly, the association between body weight and mental health impairment in women does not appear to be confined to overweight. Rather, findings from those studies that have considered associations with mental health across the full spectrum of body weight suggest the presence of U-shaped relationships, such that both underweight and overweight are associated with poorer mental health in women [3,6,7,14,21]. In other studies, only underweight has been found to be associated with mental health impairment [22,23]. However, the evidence base is small and, as with the literature relating to obesity and mental health, associations have been found to depend on the characteristics of the study population, the measures of mental health employed and whether or not the influence of potential covariates, such as physical health and chronic medical conditions, is taken into account in the analysis [5,6,8,21,24].

In any case, the observation that mental health may be impaired in underweight women would seem to present a paradox. That is, if it is accepted that impairment in women's mental health associated with obesity is due primarily to the effects of body dissatisfaction and/or eating-disordered behaviour, then it would seem reasonable to hypothesise that low body weight would tend to be associated with *better *mental health [25,26]. An ego-syntonic effect of low body weight in women - and the fact such an effect would not be expected in men - might help to explain why mental health impairment has been observed in underweight men - but not women - in some studies [1,16,27]. On the other hand, a tendency for underweight women to have lower levels of body dissatisfaction and disordered eating might be counter-balanced by the presence of a sub-group of underweight women with very high levels of body dissatisfaction and eating disorder symptoms, namely, those with anorexia nervosa or variants of this disorder not meeting formal diagnostic criteria [28].

A reading of the literature indicates that an overrepresentation of individuals with high levels of body dissatisfaction and/or eating-disordered behaviour is, in fact, the favoured explanation for the finding of mental health impairment in underweight women. Thus, Ali & Lindstrom [22] noted that body image distortion seems to be associated with underweight among young women in the industrialised world and that "anorexia and bulimia may be considered as the most severe and ultimate causes of underweight among young women" (p.324). Similarly, Ford and colleagues [9] noted that low BMI may be significantly associated with female gender and, in turn, greater weight loss goals when dieting and that lean individuals are likely to be a heterogeneous group that includes "healthy persons who exercise a lot, persons with eating disorders and clinically or subclinically sick persons" (p.26). The putative association between low body weight and body dissatisfaction/eating-disordered behaviour has been invoked as an explanation of the association between low body weight and mental health impairment in at least four other studies in which such an association has been observed [3,14,21,23] as well as in research conducted in other fields [26].

Importantly, however, measures of body dissatisfaction and/or eating disorder psychopathology were not included in any of these studies. Hence, the hypothesised associations between body weight, body dissatisfaction/eating-disordered behaviour and mental health impairment could not be tested. To our knowledge, only one epidemiological study has included some assessment of body dissatisfaction and/or eating-disordered behaviour, in addition to body weight and mental health. In a community sample of women aged 18 to 25 years, Becker et al [21] found that the lifetime prevalence of any mental disorder was higher in underweight women than in normal-weight women, even after individuals with a lifetime diagnosis of anorexia or bulimia nervosa were excluded from the underweight group. However, findings from this study are difficult to interpret because the assessment of both eating disorders and other mental health problems was confined to the presence or absence of disorders meeting formal diagnostic criteria [29,30], the number of participants meeting these criteria was small and there was no assessment of body dissatisfaction.

In sum, there appears to be little direct evidence to support - or refute - the popular notion that underweight is associated with elevated levels of body dissatisfaction and/or eating disordered behaviour in women or that such an association accounts for mental health impairment. With this in mind, the goals of the present study were as follows. First, we sought to confirm that mental health is in fact impaired in underweight women, when compared with normal-weight women. Second, we tested the hypothesis that body dissatisfaction and/or eating disordered behaviour are greater in underweight women than in normal-weight women. If both of these conditions held, then it would be possible to test the additional hypothesis that impairment in mental health among underweight women is accounted for by body dissatisfaction and/or eating-disordered behaviour.

## Methods

### Study design and participants

The research was conducted as part of the *Health and Well-Being of Female ACT Residents Study*, an epidemiological study of disability associated with eating-disordered behaviour among young adult women [19,30-36]. The study was carried out in the Australian Capital Territory (ACT) region of Australia, a highly urbanised region that includes the city of Canberra (population of 314,000 in 2002). Young women were chosen because of the comparatively high prevalence of body dissatisfaction and eating-disordered behaviour in this population [37]. All aspects of the study design and methods were approved by the ACT Human Research Ethics Committee.

A detailed account of the study methods can be found in several previous publications [19,30-36]. In brief, self-report questionnaires were initially completed by 5,255 female ACT residents aged 18 to 42 years. The questionnaire included measures of eating-disordered behaviour (including items assessing body dissatisfaction), health-related quality of life, subjective quality of life, general psychological distress, physical activity and demographic information. Demographic variables assessed included: age in years; country of birth (Australia, not Australia); first language (English, not English); marital status (married, not married); parity (no children, one or more children); main activity (employed full-time, not employed full-time); educational attainment (bachelor's degree or higher qualification completed/not completed); and possession of (private) health insurance (no, yes). Body mass index (BMI) (kg/m^**2**^) was calculated from self-reported height and weight [38].

The sample comprised approximately 10% of the total population of young adult women in the ACT and was representative of this population in terms of socio-demographic characteristics [34]. Thus, most participants were born in Australia (85.3%), had English as their first language (91.8%) and had completed 12 or more years of formal education (90.5%). Fifty-five per cent of participants were married or living as married, 43.8% had one or more children, 62.8% were employed full- or part-time, 15.6% were full-time students and 17.5% nominated home duties as their main activity.

The mean (SD) age of participants was 30.3 (7.2) years. The mean (SD) (BMI) among the 4,892 (93.1%) participants who provided details of both height and weight was 24.5 (5.3) kg/m^2^. Reflecting the study aims, participants in the present study were the 231 women (4.7%) who were underweight (BMI < 18.5) and 2,976 women (60.8%) who were normal-weight (18.5 ≤ BMI < 25.0) according to the conventional classification [39]. Findings relating to the associations between obesity, eating-disordered behaviour and mental health have been reported elsewhere [19,30,36].

### Study measures

#### Body dissatisfaction and eating disordered behaviour

Eating-disordered behaviour was assessed using the Eating Disorder Examination Questionnaire (EDE-Q) [40], a widely-used, 36-item, self-report measure that focuses on the occurrence and frequency of key eating disorder attitudes and behaviours during the past 28 days. Subscale scores - relating to dietary intake/restraint, concerns about eating and concerns about weight or shape - and a global score, are derived from 22 items addressing attitudinal features [34]. Scores on each (item and) scale range from 0 to 6, with higher scores indicating higher symptom levels. Remaining items of the EDE-Q assess the occurrence and frequency of specific eating disorder behaviours, namely, binge eating, self-induced vomiting, misuse of laxatives or diuretics, extreme dietary restriction and excessive exercise. These items do not contribute to subscale scores.

Two of the EDE-Q (Weight/Shape Concerns subscale) items specifically address body dissatisfaction, namely, "How dissatisfied have you felt about your weight" and "How dissatisfied have you felt about your shape". The average of scores on these 2 items, which were highly correlated (r = 0.89), was used as a measure of body dissatisfaction in the present study [41].

Whereas the EDE-Q global score provided a continuous measure of eating disorder psychopathology, eating disorder "cases" were identified using an operational definition informed by our previous research, namely, the occurrence of extreme weight or shape concerns in conjunction any regular eating disorder behaviour [38,30]. For binge eating, self-induced vomiting, and purging behaviours, "regular" was defined as "at least weekly", whereas regular extreme dietary restriction and excessive exercise were recognised if these behaviours occurred, on average, 3 or more times per week [34]. Although, in the absence of interview assessment, participants meeting these criteria should be viewed as "probable" rather than "true" cases, the criteria have been found to identify individuals with high levels of eating disorder psychopathology and functional impairment [38,30].

### Mental health

#### Health-related quality of life

Health-related quality of life was assessed with the Medical Outcomes Study (12-item) Short-Form disability scale (SF-12) [42]. Items of the SF-12 are summarised into two weighted scales (Physical Component Summary scale, PCS; Mental Component Summary scale, MCS), designed to assess physical and mental health impairment. Each scale is scored to have a mean of 50 and standard deviation of 10 (in the US population), with lower scores indicating higher levels of impairment. The SF-12 has very good psychometric properties, including demonstrated validity in the Australian population [42,43]. PCS items include "Does your health now limit you in moderate activities, such as moving a table, vacuuming or playing golf?" and "During the past four weeks, were you limited in the kind or work or other activities undertaken as a result of your physical health?", whereas MCS items include "During the past four weeks have you accomplished less than you would like as a result of any emotional problems?" and "During the past four weeks how much of the time have you felt calm and peaceful"? In the present study, the SF-12 MCS was the outcome of interest whereas physical health, as assessed by the SF-12 PCS, was included as a covariate. Cronbach's alpha was 0.82 for the total scale and 0.80 for the 6 items comprising the MCS.

#### Subjective quality of life

Subjective quality of life was assessed using the World Health Organization Brief Quality of Life Assessment Scale (WHOQOL-BREF) [44,45], a 26-item measure yielding scores on each of four domains relating to the individual's subjective evaluation of their physical health, environmental health, psychological health and social relationships. Items are scored on a five-point, Likert-type scale, with scores of ''1'' and "5" indicating, respectively, extreme dissatisfaction and extreme satisfaction. Only the Psychological Functioning (QOL-P) subscale, which can be viewed as a measure of perceived satisfaction with key aspects of emotional well-being, was considered in the present study. Items of the QoL-P include "To what extent do you feel your life to be meaningful"? and "How satisfied are you with yourself?' One of the (6) items comprising the QOL-P, which addresses satisfaction with "bodily appearance", was excluded when calculating the scale score. Cronbach alphas for the 5- and 6-item scales were, respectively, 0.80 and 0.81.

#### General psychological distress

General psychological distress was assessed with the Kessler Psychological Distress Scale (K-10), a 10-item self-report measure designed for use in general population surveys [46]. In Australia it is also used as an outcome measure among individuals treated within mental health services [47]. The frequency (during the past four weeks) of each of 10 symptoms - relating to anxiety and depressive mood - is measured on a scale from one to five, such that total scores range from 10 to 50 with lower scores indicating higher symptom levels. This coding method was employed - i.e. in preference to an alternative method in which lower scores indicate lower symptoms levels [47] - so that lower scores would indicate poorer mental health for all 3 mental health measures. Findings from the Australian National Survey of Mental Health and Well-Being suggested that individuals scoring in the extreme range (≤ 30) have a high probability of meeting diagnostic criteria for an anxiety or affective disorder according to interview assessment [47]. Cronbach's alpha in present study was 0.91.

#### Physical activity

In addition to the questions assessing the use of exercise as a means of weight control (included in the EDE-Q), three questions were included that assessed the frequency of mild, moderate and hard exercise during the past four weeks [35]. Based on these questions, a dichotomous variable was created such that participants who reported any of the three forms of exercise on average at least 3 times per week during the past four weeks were considered to be regular exercisers.

#### Statistical analysis

Loess curves were used to examine the associations between BMI, as a continuous variable, and each measure of mental health - SF-12 MCS, QOL-P and K-10 - in the total sample (n = 4,892). Loess, which stands for locally weighted scatterplot smoothing, is a method for fitting a curve to a scatter plot that provides a graphical representation of the relationship between two variables without making any a priori assumptions about the form of that relationship [48].

Independent-samples t-tests were used to compare scores on continuous variables, namely, age, BMI, body dissatisfaction, EDE-Q subscale scores and scores on the SF-12 PCS and MCS, QOL-P and K-10, between underweight and normal weight participants, whereas chi-square tests were used to compare groups on categorical outcomes, namely, demographic characteristics, the occurrence of specific eating disorder behaviours, the occurrence of regular physical activity and the prevalence of probable eating disorder cases. Bivariate correlations were calculated using the Pearson correlation coefficient. Linear regression models [49] were used to test the hypothesis that impairment in mental health associated with low body weight, where this was observed, was accounted for by body dissatisfaction and/or eating-disordered behaviour.

A significance level of 0.05 was employed for all tests, all tests were two-tailed and all analysis was conducted using SPSS version 17.0. For analyses involving the SF-12, both the standard scoring method, employing factor scores derived by means of orthogonal rotation, and an alternative method, employing factor scores derived by means of oblique rotation, were employed [50]. Since the main findings were unchanged, only findings based on the standard scoring method are reported.

## Results

**Figure 1:**
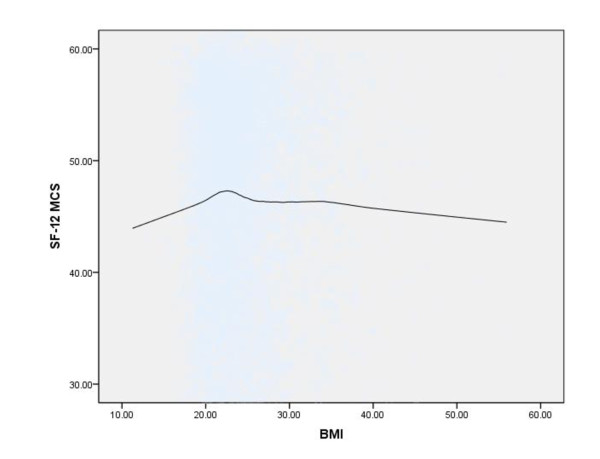
**Loess curve showing the association between body mass index (BMI) (kg/m^2^) and mental health functioning, as measured by the Medical Outcomes Study Short Form Mental Component Summary Scale (SF-12 MCS), in the total sample (n = 4,892) (Note: lower scores on the SF-12 MCS indicate greater mental health impairment)**.

**Figure 2:**
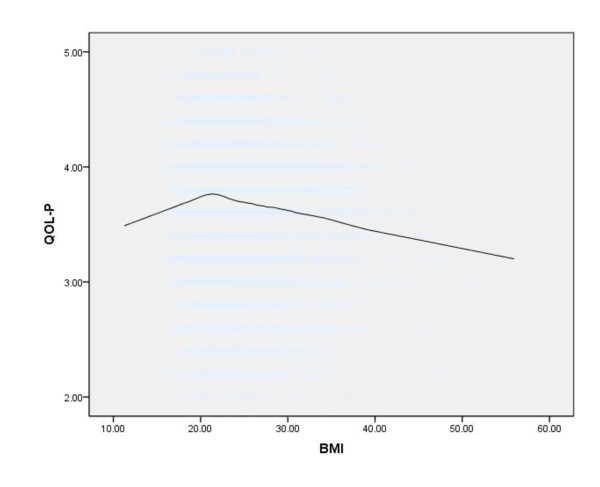
**Loess curve showing the association between body mass index (BMI) (kg/m^2^) and subjective mental health, as measured by the WHOQOL-BREF Psychological Health subscale (QOL-P), in the total sample (n = 4,892) (Note: lower scores on the QOL-P indicate greater mental health impairment)**.

**Figure 3:**
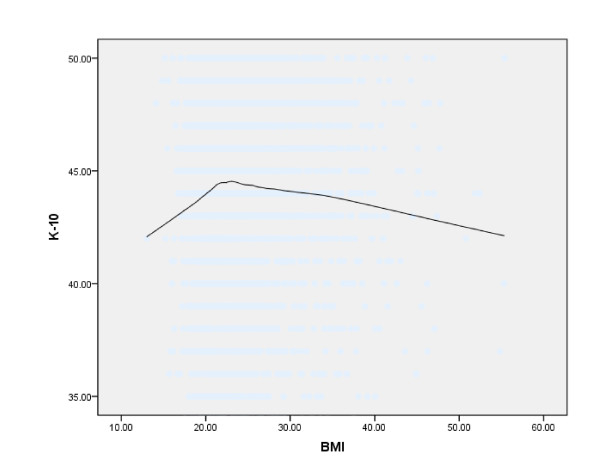
**Loess curve showing the association between body mass index (BMI) (kg/m2) and general psychological distress, as measured by the Kessler Psychological Distress Scale (K-10) in the total sample (n = 4,892) (Note: lower scores on the K-10 indicate greater mental health impairment)**.

Figures 1, 2, and 3 show Loess curves of the relationships between BMI and scores on the SF-12 MCS, QOL-P, and K-10, respectively. As can be seen, both very low and very high body weights were associated with mental health impairment and this was the case for all 3 measures.

As would be expected, moderate to high positive correlations were observed between the different measures of mental health (MCS, QOL-P: 0.71; QOL-P, K-10: 0.72; MCS, K-10: 0.76). Further, body dissatisfaction was highly correlated with overall levels of eating disorder psychopathology as measured by the EDE-Q Global score (r = 0.82).

Underweight women were less likely to be married (24.2% vs 37.2%; × = 15.51, p < 0.01), less likely to have one or more children (30.1% vs 39.9%; × = 8.52.5, p < 0.01), less likely to have completed tertiary studies (29.5% vs 41.7%; × = 12.95, p < 0.01) and less likely to have private health insurance (50.5% vs 60.1%; × = 7.14, p < 0.01) than normal weight-women, whereas the groups did not differ with respect to employment, country of birth or first language (all p > 0.05).

Comparisons between underweight and normal-weight participants on continuous variables are shown in Table 1. As can be seen, underweight women were younger and had lower scores (indicating higher levels of impairment) on the SF-12 PCS and on all 3 measures of mental health, than normal-weight women, although differences on the SF-12 MCS did not reach statistical significance. It is also apparent that underweight women had lower levels of body dissatisfaction and lower scores on each of the EDE-Q subscales than normal-weight women.

**Table 1:** Mean (SD) age, BMI and scores on measures of body dissatisfaction, eating disorder features and mental health for underweight and normal-weight women

	Underweight (n = 231)	Normal-Weight (n = 2976)			
	**Mean (SD)**	**Mean (SD)**	**t**	**p**	***d***

Age	26.87 (7.31)	29.71 (7.25)	-5.72	< 0.01	0.39
BMI (kg/m^2^)	17.52 (0.97)	21.90 (1.71)	-38.55	< 0.01	3.15
Eating Disorder Examination Subscales					
Body dissatisfaction	1.38 (1.48)	2.15 (1.79)	-6.31	< 0.01	0.47
Restraint	0.75 (1.29)	1.15 (1.33)	-4.38	< 0.01	0.31
Eating concern	0.45 (0.90)	0.57 (0.88)	-1.98	< 0.05	0.14
Weight/Shape concern	1.18 (1.24)	1.78 (1.45)	-6.14	< 0.01	0.44
Global score	0.83 (1.05)	1.24 (1.12)	-5.28	< 0.01	0.38
SF-12 PCS^i^	49.74 (8.76)	50.87 (7.70)	-2.08	< 0.05	0.14
SF-12 MCS^ii^	44.83 (10.78)	46.06 (10.49)	-1.67	0.09	0.12
QOL-P^iii^	3.59 (0.63)	3.69 (0.60)	-2.28	< 0.05	0.16
K-10^iv^	41.85 (6.67)	43.27 (5.83)	-3.25	< 0.01	0.23

i SF-12 Physical Component Summary scale.

ii SF-12 Mental Component Summary scale.

iii WHOQOL-BREF Psychological Functioning subscale.

iv Kessler Psychological Distress Scale.

There were no differences between groups with respect to the occurrence of eating disorder behaviours (all p > 0.05), nor with respect to the prevalence of probable eating disorder cases (underweight: 3.5%; normal-weight: 5.8%; × = 2.27, p = 0.13). However, underweight women were less likely to be regular exercisers than normal-weight women (47.7% vs 56.6%; × = 5.25, p < 0.05).

Since underweight was associated with lower, rather than higher, levels of body dissatisfaction and eating disorder psychopathology, there was no basis on which to proceed with formal tests of the hypothesis of mediation. Post-hoc analysis was conducted, however, in order to determine which variables (other than body dissatisfaction and eating-disordered behaviour) might have accounted for the observed association between low body weight and mental health impairment and to elucidate the comparative importance of different variables in accounting for mental health impairment among underweight participants.

For the first analysis, hierarchical linear regression was used to determine if differences between groups in mental health impairment remained after controlling for potential covariates, namely, those variables that differed between groups in bivariate analysis. A dichotomous variable indicating weight status (underweight, normal-weight) was used in place of BMI for this analysis. A similar method was employed for the second analysis, except that all variables were entered simultaneously, body dissatisfaction and eating-disordered behaviour (as measured by the EDE-Q global score) were included and weight status was replaced with BMI.

Results of the first analysis are summarised in Table 2. As can be seen, the association between weight status and scores on the K-10 remained significant (p = 0.04) after controlling for demographic variables and approached significance (p = 0.07) after physical health and physical activity were added to the model. For the QOL-P, by contrast, the inclusion of demographic variables alone resulted in the initial contribution of weight status becoming non-significant. Similarly, for the SF-12 MCS, weight status no longer approached significance after demographic variables were included.

**Table 2:** Multiple linear regression analysis of the association between weight status (underweight, normal-weight) and each measure of mental health (SF-12 MCS, QOL-P and K-10) with and without the inclusion of additional covariates (demographic variables; physical health and physical activity) (n = 3,207)

		SF-12 MCS				QOL-P				K-10		
**Model**	**Covariates**	**B**	**p**	**R^2^**		**B**	**p**	**R^2^**		**B**	**p**	**R^2^**

1	Weight status^i^	1.229	0.095	0.001		0.095	0.023	0.002		1.420	0.001	0.004
2	Weight status, demographic variables^ii^	0.330	0.677	0.028		0.057	0.205	0.023		0.896	0.039	0.043
3	Weight status, demographic variables, physical health^iii^, physical activity	0.437	0.583	0.049		0.034	0.455	0.043		0.799	0.072	0.062

i Underweight = 0; normal-weight = 1.

ii Age, marital status parity, educational attainment, private health insurance.

iii As measured by the SF-12 PCS.

Results of the second set of analyses are summarised in Table 3. As can be seen, body dissatisfaction was the strongest - and only strong - predictor of greater mental health impairment and this was the case for all 3 dependent variables.

**Table 3:** Multiple linear regression analysis of variables associated with each measure of mental health (SF-12 MCS, QOL-P and K-10) among underweight women (n = 231)

	SF-12 MCS		QOL-P		K-10	
**Covariates**	**β**	**p**	**β**	**p**	**β**	**p**

Age	.017	.872	-.011	.912	-.019	.856
BMI	-.070	.344	-.076	.289	-.094	.206
Marital status	.102	.245	.016	.855	.052	.552
Parity	-.016	.872	-.019	.842	.060	.548
Employment	-.103	.197	-.030	.695	-.083	.300
Education	-.063	.454	.024	.770	.004	.958
Country of birth	.013	.886	-.095	.266	-.056	.529
First language	.083	.361	.208	.018	.151	.097
Health insurance	.064	.387	.098	.167	.153	.040
Physical health	-.127	.082	.179	.011	.086	.229
Physical activity	.070	.376	.085	.271	.016	.844
Body dissatisfaction	-.400	.000	-.513	.000	-.478	.000
Eating disorder psychopathology	-.023	.817	.039	.683	.077	.445

## Discussion

### Summary of main findings

We sought to evaluate the hypothesis that mental health impairment in underweight women, where this occurs, is due to an association between low body weight and elevated levels of body dissatisfaction and/or eating-disordered behaviour. To this end, subgroups of underweight and normal-weight women recruited from a large, general population sample were compared on measures of body dissatisfaction, eating-disordered behaviour and mental health. There were two main findings. First, underweight women had significantly greater impairment in mental health than normal-weight women, even after controlling for differences in demographic characteristics and physical health. Second, there was no evidence that higher levels of body dissatisfaction or eating-disordered behaviour accounted for this difference. Rather, underweight women had significantly lower levels of body dissatisfaction and eating-disordered behaviour than normal-weight women. There was also no evidence that greater mental health impairment in the underweight group may have been due to the presence of a small number of individuals with very high levels of eating disorder psychopathology, namely, individuals likely to have an eating disorder.

### Study implications

The primary implication of the present study is that mental health impairment in underweight women, where this occurs, is unlikely to be accounted for by an association between low body weight and elevated levels of body dissatisfaction or eating-disordered behaviour. Rather, body dissatisfaction and eating disordered behaviour appear to be comparatively uncommon among underweight women. Interestingly, however, body dissatisfaction was still strongly predictive of poor mental health in multivariable analysis conducted within the underweight group. Taken together, these findings suggest not only that higher levels of body dissatisfaction or eating-disordered behaviour among underweight women do not account for mental health impairment, but also that lower levels of body dissatisfaction and/or eating disordered behaviour among underweight women may counterbalance, to some extent, mental health impairment due to other factors.

Consistent with findings from other recent epidemiological studies [7,14], the prevalence of underweight was low among women in the present study, less than 5%. Given current concern surrounding the high prevalence of obesity in industrialised nations, research addressing the impact of underweight on mental health has not been a priority. Indeed, underweight individuals have often been excluded in studies of the association between body weight and mental health due to concerns that high levels of impairment among underweight individuals might complicate interpretation of comparisons between obese and non-obese individuals [3,5]. Similar concerns have arisen in research addressing the association between obesity and mortality [24]. However, it is important to critically evaluate the validity of anecdotal evidence, particularly when there are implications for public health practice. For example, Ali & Lindstrom [22] noted that interventions to improve psychological health in underweight women would need to deal with the body norms/image messages disseminated in the popular media. The present findings argue against this view. The findings do suggest, however, that women who are very underweight - like those who are very overweight - are a vulnerable group, being at increased risk of impairment in both physical and mental health.

We can only speculate as to why the notion that low body weight is associated with body dissatisfaction and/or eating-disordered behaviour is so widely accepted when there is so little evidence to support it. There may be poor understanding of the epidemiology of eating-disordered behaviour among researchers not familiar with this literature, for example, low awareness of the fact that eating disorders characterised by normal or above-average body weight far outnumber those characterised by low body weight [29]. There may also be a tendency for public health researchers to generalise from the clinical/hospital setting, in which individuals presenting with the combination of low body weight and extreme concerns about weight or shape are more conspicuous [51]. In any case, our findings suggest that there is a need to address the misconception that low body weight is associated with body dissatisfaction and/or eating-disordered behaviour in unselected samples.

### Study limitations and other methodological considerations

Several limitations of the present study should be noted. First, some potentially important covariates were not assessed. In particular, there was no assessment of smoking or of chronic medical conditions, both of which may be associated with low body weight and/or mental health impairment [6,7,22,24]. The higher levels of mental health impairment observed in underweight women might also have been due to the presence of a small number of individuals with very high symptom levels, namely, those with anxiety, affective, substance use or other mental disorders [6,7]. Interview assessment would be required to test this hypothesis. Our goal was to test the hypothesis that body dissatisfaction/eating-disordered behaviour mediates the association between low body weight and mental health impairment, rather than to examine factors associated with impairment.

Second, approximately 40% of individuals approached to participate in the study chose not to return a completed questionnaire and individuals with anorexia or variants of anorexia may be over-represented in this subgroup [52]. To the extent that a bias of this kind occurred, both the extent of mental health impairment in the underweight group and the role of body dissatisfaction/eating disorder psychopathology in accounting for this impairment may have been underestimated. Individuals with other mental disorders may also have been over-represented among non-respondents [53]. However, these observations do not change the fact that, in the present study, greater mental health impairment was observed among underweight women despite these women having lower levels of body dissatisfaction and eating disorder psychopathology than normal-weight women.

Third, the present findings necessarily apply to underweight defined as a BMI of < 18.5 kg/m^2^. Although this criterion is widely accepted, it is nevertheless arbitrary and different findings may have been observed had a more or less stringent operation definition of low body weight been employed [1,22]. In addition, BMI was calculated based on self-reported height and weight in the present study. However, we found very good agreement between BMI based on self-reported height and weight and BMI derived from actual (measured) height and weight in pilot work [38].

Fourth, the present findings necessarily apply to younger women from an urbanised, affluent region. This population was appropriate for an initial study because the hypothesis that impairment in mental health associated with low body weight is due to body dissatisfaction and/or eating-disordered behaviour has been proposed primarily in relation to young women from industrialised nations [22,26]. As suggested previously, it may make more sense to consider the role of body dissatisfaction in relation to mental health impairment in underweight men, given that underweight males are more likely to be dissatisfied with their bodies than normal-weight males and given that the prevalence of body dissatisfaction and its impact on mental health may be increasing in males [1,20,27,54].

Some comment is warranted concerning the treatment of body dissatisfaction and eating-disordered behaviour as distinct constructs. The key distinction between individuals with extreme weight or shape concerns and individuals with eating disorders is the regular occurrence of one or more eating disorder (i.e. binge eating or extreme weight-control) behaviours. Since extreme weight or shape concerns in the absence of eating disorder behaviours are more common than the combination of concerns and behaviours, it is not surprising that body dissatisfaction emerged as the stronger predictor of impairment among underweight participants. But it needs to be remembered that there is extensive overlap between these constructs in general population samples [41].

Finally, since this was a cross-sectional study, the usual caveats concerning the direction of any observed associations apply [4,55]. The available evidence from longitudinal studies suggests that associations between body dissatisfaction/eating disordered behaviour and mental health impairment are likely to be bidirectional [56-58]. Notable strengths of the present research were the recruitment of a large, general population sample of women, comprehensive assessment of eating-disordered behaviour and the inclusion of three different measures of mental health.

## Conclusions

To conclude, the findings of the present study suggest that mental health impairment in underweight women, where this occurs, is unlikely to be due to higher levels of body dissatisfaction or eating-disordered behaviour. Rather, lower levels of body dissatisfaction and eating-disordered behaviour among underweight women may counterbalance, to some extent, impairment due to other factors. The findings also suggest that women who are very underweight are a vulnerable group, being at increased risk of impairment in both physical and mental health.

## Ethics approval

The research was conducted with the approval of the ACT Human Research Ethics Committee.

## Competing interests

The authors declare that they have no competing interests.

## Authors' contributions

JM was responsible for the design and conduct of the research as well as data processing, data analysis and manuscript preparation. BR, PH and CO contributed to the design and conduct of the research and to critical revision of an earlier version of the manuscript. BR contributed to data analysis and interpretation. All authors read and approved the final manuscript.

## Pre-publication history

The pre-publication history for this paper can be accessed here:

http://www.biomedcentral.com/1471-2458/11/547/prepub
